# Impact of the H275Y and I223V Mutations in the Neuraminidase of the 2009 Pandemic Influenza Virus In Vitro and Evaluating Experimental Reproducibility

**DOI:** 10.1371/journal.pone.0126115

**Published:** 2015-05-20

**Authors:** Eric G. Paradis, Lady Tatiana Pinilla, Benjamin P. Holder, Yacine Abed, Guy Boivin, Catherine A.A. Beauchemin

**Affiliations:** 1 Department of Physics, Ryerson University, Toronto, ON, Canada; 2 Infectious Disease Research Centre, CHUQ-CHUL and Laval University, Québec, QC, Canada; St. Jude Children's Research Hospital, UNITED STATES

## Abstract

The 2009 pandemic H1N1 (H1N1pdm09) influenza virus is naturally susceptible to neuraminidase (NA) inhibitors, but mutations in the NA protein can cause oseltamivir resistance. The H275Y and I223V amino acid substitutions in the NA of the H1N1pdm09 influenza strain have been separately observed in patients exhibiting oseltamivir-resistance. Here, we apply mathematical modelling techniques to compare the fitness of the wild-type H1N1pdm09 strain relative to each of these two mutants. We find that both the H275Y and I223V mutations in the H1N1pdm09 background significantly lengthen the duration of the eclipse phase (by 2.5 h and 3.6 h, respectively), consistent with these NA mutations delaying the release of viral progeny from newly infected cells. Cells infected by H1N1pdm09 virus carrying the I223V mutation display a disadvantageous, shorter infectious lifespan (17 h shorter) than those infected with the wild-type or MUT-H275Y strains. In terms of compensating traits, the H275Y mutation in the H1N1pdm09 background results in increased virus infectiousness, as we reported previously, whereas the I223V exhibits none, leaving it overall less fit than both its wild-type counterpart and the MUT-H275Y strain. Using computer simulated competition experiments, we determine that in the presence of oseltamivir at doses even below standard therapy, both the MUT-H275Y and MUT-I223V dominate their wild-type counterpart in all aspects, and the MUT-H275Y outcompetes the MUT-I223V. The H275Y mutation should therefore be more commonly observed than the I223V mutation in circulating H1N1pdm09 strains, assuming both mutations have a similar impact or no significant impact on between-host transmission. We also show that mathematical modelling offers a relatively inexpensive and reliable means to quantify inter-experimental variability and assess the reproducibility of results.

## Introduction

The 2009–2010 influenza season saw the emergence of a new influenza strain, H1N1pdm09, that reached pandemic status and was declared a global health concern [1]. As the prior seasonal H1N1 strain (A/Brisbane/59/2007) had developed a nearly complete resistance to oseltamivir [2], the H1N1pdm09 strain was monitored for any such emerging resistance. The resistance in the seasonal strain, due to a histidine-to-tyrosine mutation at position 275 of the neuraminidase (NA) protein (H275Y) and subsequent permissive mutations [3, 4], raised concern about similar mutations occurring within the pandemic strain.

Initial studies showed that the H1N1pdm09 strain did not bear the H275Y mutation and was susceptible to NA inhibitors [5]. However in recent years some resistance has been reported [6–8], and subsequent analysis revealed the presence of the H275Y mutation in a large number of these cases [9–12]. Experimental measurements of IC_50_ values revealed that the H275Y mutation reduces susceptibility to both oseltamivir (980-fold for A/Québec/144147/09) and peramivir (660-fold) [13–16]. Comparative studies and competition trials have demonstrated that the H275Y mutation is accompanied by only a minor reduction in fitness [15, 16], and evidence of community transmission has recently been observed [12, 17]. In a previous publication [18], we identified a set of experimental assays and a mathematical modelling approach that together determine the key viral replication parameters characterizing the particular strain in question. This analysis revealed that the primary effects of the H275Y substitution were an increase of the initial eclipse period and a decrease of the viral burst size, with little decrease to overall fitness.

An isoleucine-to-valine mutation at residue 223 (I223V) of the NA protein also reduced susceptibility to oseltamivir (6-fold), peramivir (3-fold), and zanamivir (2-fold) [13]. The I223V [19] and isoleucine-to-arginine (I223R) [20, 21] mutations have been detected in patients treated with oseltamivir, suggesting the possible emergence of a viable resistant strain through an I223 mutation. Fitness studies of mutations at residue 223 have produced varied results, from reduced viral titers and plaques sizes for the I223R mutant [22] to improved replication for both I223R and I223V [13]. Another study of the I223R mutant observed a 6–12 hour delay of initial viral replication with MDCK-2,6 (SIAT-1) cells [23].

In this report, we apply a mathematical model introduced in previous work [18], to analyze a set of experiments with the H1N1pdm09 wild-type strain and its I223V single-mutant counterpart. We evaluate the impact of this I223V mutation on the fitness of the H1N1pdm09 influenza strain by analyzing the viral load curves and extracting the key biological parameters characterizing the replicative fitness. We also compare these extracted parameters to those recovered from our previous work [18] to assess the fitness of both the H275Y and I223V single-mutants, relative to the H1N1pdm09 influenza strain and to one another. Simulated competition experiments based on the extracted parameters are also conducted to provide an efficient means of comparing relative fitness of mutant strains across experiments both in the presence and absence of antiviral selective pressure.

We also investigate the issue of experimental reproducibility in light of inter-experimental variability and propose a new methodology to tackle the issue. There are growing concerns over the lack of reproducibility of results in the health sciences [24–30]. Due to inter-experimental variability, viral load curves (quantified via cell culture or qPCR) from one experimental data set cannot normally be compared to those from a distinct experimental data set performed at a later time, even within the same laboratory, following the same procedure. Here, we compare the characteristics of the same strain (a recombinant of the H1N1pdm09 A/Québec/144147/09) across experiments and evaluate the impact of inter-experimental variability on inferred strain properties. In particular, we show how our modelling analysis enables us to test reproducibility by identifying and quantifying inter-experimental variability—including a determination of which parameters are susceptible to this variability and which are not. We show that while any one strain’s parameters can vary significantly between experiments, inter-experimental variations appear to affect different strains similarly, such that the changes in parameters of one strain relative to another, may be conserved between experiments: an important new finding. We demonstrate how mathematical modelling can be used to bridge the gap across experiments, namely by expressing a strain’s parameters in terms of fold-change relative to that of an unchanging reference strain (a reassortant monitored to contain no mutation) to be used as the standard curve in separate experiments.

## Results

### Effect of the I223V mutation in the H1N1pdm09 background

**Fig 1:**
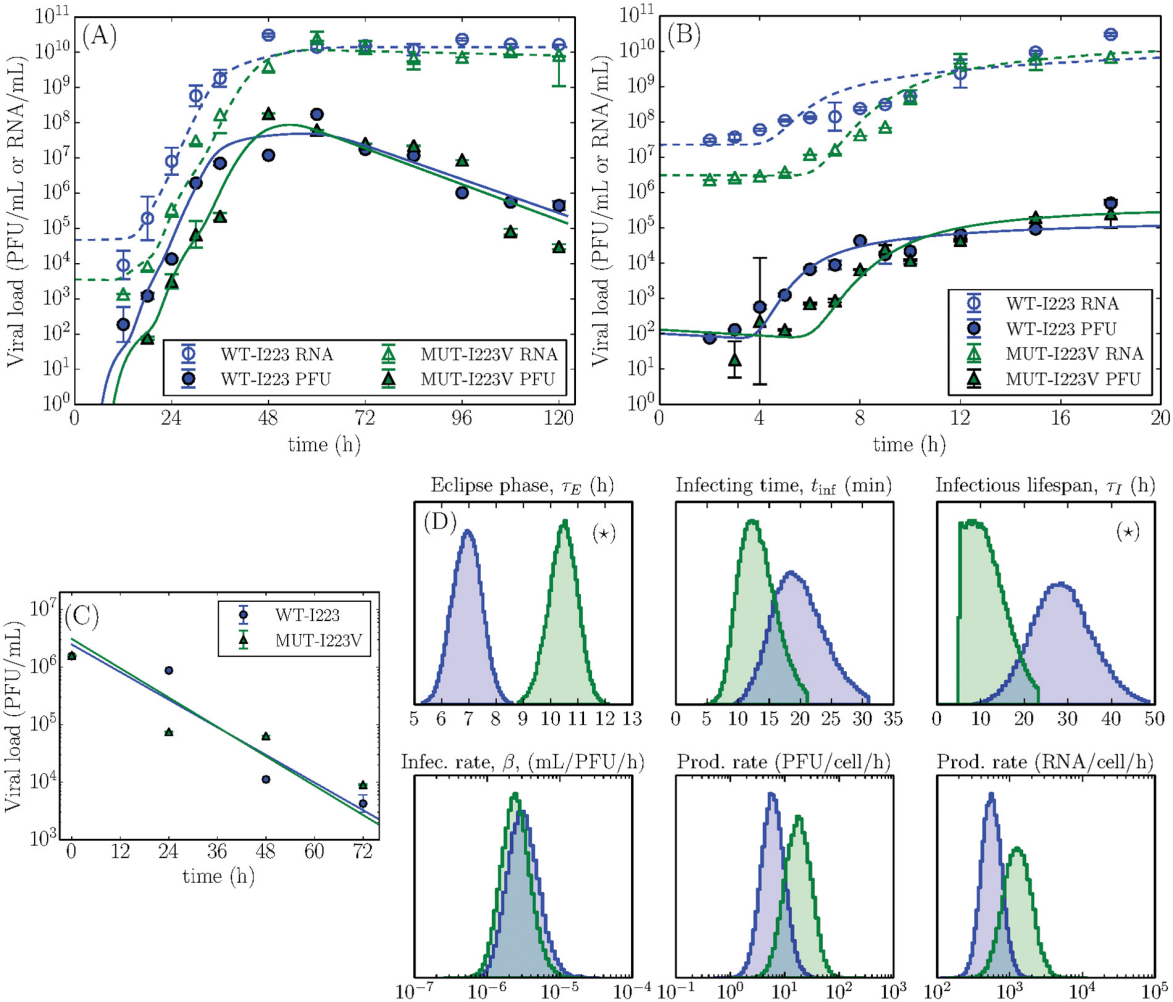
Experimental data and extracted parameters characterizing virus replication fitness. Measured H1N1pdm09 WT-I223 (blue circles) and MUT-I223V (green triangles) viral load concentrations for the (A) multiple-cycle, (B) single-cycle, and (C) mock-yield assays. The points represent the geometric mean and their error bars correspond to the standard deviation of the experimental data points collected in triplicate at each time point. The lines were produced using Eq (1) with the median parameter values presented in Table 1 solved as described in Methods. (D) Probability density functions of the key virus replication parameters with statistically significant differences indicated with a (⋆).

The parameter distributions extracted from the MCMC analysis (see Methods) for the H1N1pdm09 strain A/Québec/144147/09 (WT-I223) and its mutant counterpart (MUT-I223V) are presented in Table 1, and the fit of the model to the data is presented in Fig 1. We find that the MUT-I223V single mutant in the H1N1pdm09 background has no statistically significant effect on most viral replication parameters, with two exceptions. The MUT-I223V has an eclipse period (*τ*_*E*_) which is 3.6 h longer (10.5 h vs 6.9 h, *p* < 0.001), and an infectious lifespan (*τ*_*I*_) 17 h shorter (11 h vs 28 h, *p* = 0.04) than that of its H1N1pdm09 WT-I223 counterpart. Both of these differences, namely the increase in *τ*_*E*_ and decrease in *τ*_*I*_, negatively affect the virus replicative fitness. For example, the increase in eclipse time is compounded over several viral infections cycles in the MC assay, and produces a ∼ 8h delay in the time for the viral titer to reach 90% of its log-scaled peak value. This is in agreement with previous work that observed a 6h–12h delay in the initial viral replication for a mutation at this same residue [23]. In Fig 1A and 1B, the total viral load (RNA/mL) plateau in both the MC and SC assays are equivalent for WT-I223 and MUT-I223V. This plateau occurs in both assays as infected cells begin to die in large numbers and the total virus concentration ceases to increase. Since the rate of total virus decay over the time scale of these experiments is negligible, the level of this plateau therefore represents the sum of all virions produced over the course of the infection. Observing the same plateau for both viruses indicates that the WT-I223 and MUT-I223V have the same virus burst size (*p*_RNA_ ⋅ *τ*_*I*_). Indeed, we computed a viral burst size of 6.5103RNA/cell for WT-I223 and 5.7103RNA/cell for MUT-I223V, a difference that is not statistically significant (*p* = 0.76). Although the MUT-I223V is capable of attaining high viral loads, its longer eclipse delay and shorter infectious lifespan relative to its wild-type counterpart in the H1N1pdm09 background puts it at a slight fitness disadvantage over the wild-type in the absence of pressure by oseltamivir therapy.

**Table 1:** Viral kinetics parameters for H1N1pdm09 WT and MUT-I223V*.

Parameter	WT-I223 Median [95% CI]	MUT-I223V Median [95% CI]	(I223 vs. V223) Significance
Eclipse period, *τ*_*E*_ (h)	6.9 [5.9–8.0]	10.5 [9.4–11.4]	***p* < 0.001**
Infecting time, *t*_infect_ (min)	19.5 [12.5–29.5]	12.9 [8.2–20.2]	*p* = 0.20
Infectious lifespan, *τ*_*I*_ (h)	28 [16–43]	11 [5–23]	***p* = 0.04**
Virion decay rate, *c*_PFU_ (h^−1^)	0.093 [0.070–0.119]	0.099 [0.077–0.122]	*p* = 0.76
Total prod. rate, *p*_RNA_ (RNA/cell/h)	560 [300–1060]	1270 [550–2930]	*p* = 0.13
Infectious prod. rate, *p*_PFU_ (PFU/cell/h)	6.0 [2.3–16.2]	17.5 [5.9–49.4]	*p* = 0.15
Virus infectiousness, *β* (mL/PFU/h)	3.1 × 10^−6^ [1.2–8.7]	2.4 × 10^−6^ [1.0–6.4]	*p* = 0.73
Inoculum infectiousness, $\frac{V_{\text{PFU}}(0)}{V_{\text{RNA}}(0)}$, $\left(\frac{\text{PFU}}{\text{RNA}}\right)$	3.8 × 10^−6^ [0.9–14.3]	36 × 10^−6^ [11–120]	***p* = 0.02**
Multiplicity of infection (MOI)	8.4 × 10^−7^ [2.3–29.3]	3.9 × 10^−7^ [1.2–12.4]	*p* = 0.38
Prod. infectivity ratio, $\frac{p_{\text{PFU,SC}}}{p_{\text{PFU,MC}}}$	3.4 × 10^−3^ [1.2 –9.4]	3.8 × 10^−3^ [1.2–11.6]	*p* = 0.89

* Median parameter values for H1N1pdm09 WT-I223, MUT-I223V, with 95% confidence intervals (CI) from MCMC analysis. Significance of parameter differences between the WT-I223 and MUT-I223V strain are provided as *p*-values.

**Fig 2:**
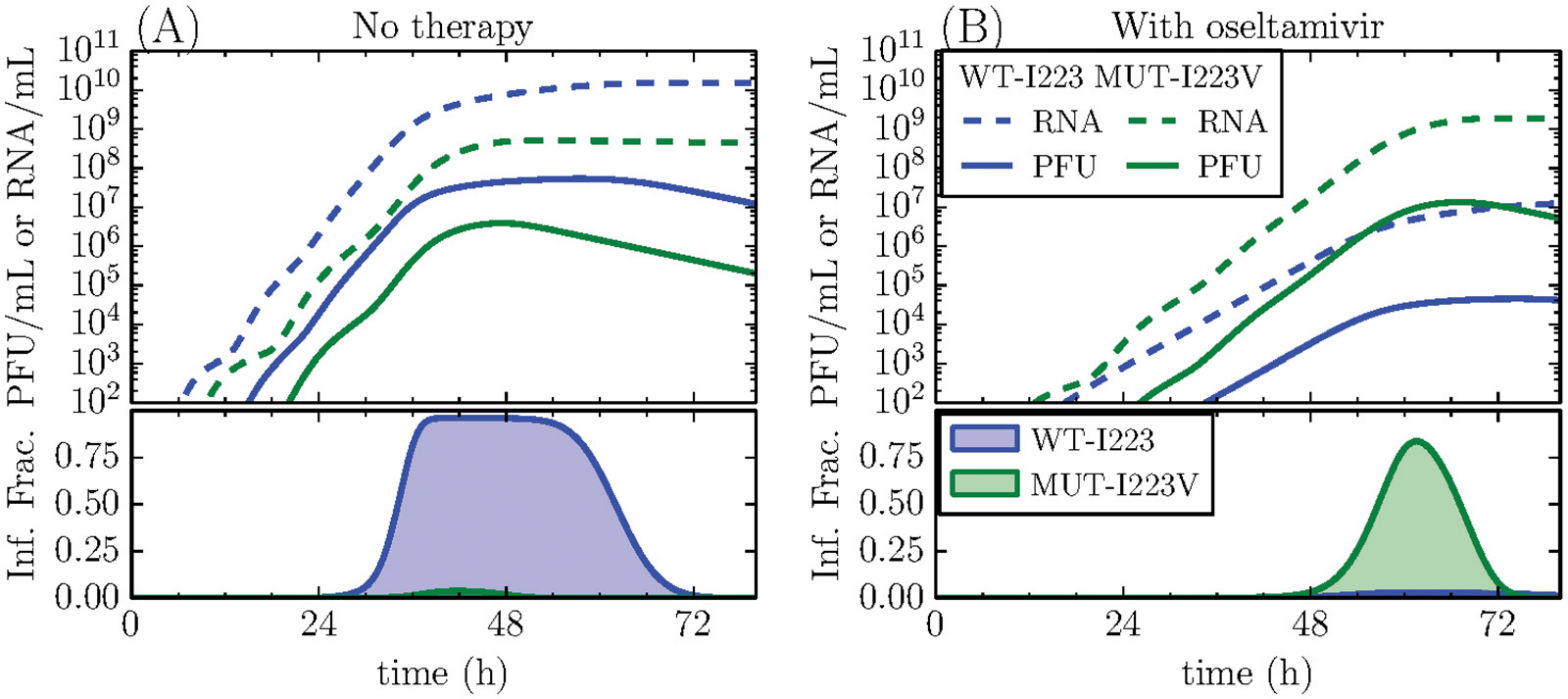
Simulated competition between the WT-I223 and MUT-I223V strains. The infectious (PFU/mL) and total (RNA/mL) viral load (top), and the fraction of infectious cells infected by each strain (bottom) are shown. The curves are produced using the median values of the extracted MCMC distributions. (A) In the absence of therapy, the mutant is disadvantaged due to its longer eclipse phase (*τ*_*E*_), and shorter infectious lifespan (*τ*_*I*_). (B) In the presence of oseltamivir, the mutant gains the fitness advantage. The quantitative gains or losses of each strain along with statistical significance are provided in the Appendix.

We also directly compare the fitness of the wild-type and single-mutant MUT-I223V strains by performing a simulated competition experiment based on the results of the MCMC analysis. Fig 2A shows the results of this simulation, where a low viral titer of equal amounts for both viral strains (25PFU each) competes to infect a population of 106cells, wherein we assume co-infection is not possible (see Methods for details). In previous work [18]—where we used the same mathematical model and the same type of experimental data—we verified that our model and approach correctly predict the course and outcome of these simulated competition experiments. Therefore, we do not repeat these validation steps here. The simulation results are shown in terms of infectious (PFU/mL) and total (RNA/mL) virus concentration, and fraction of cells infected by each strain (as detailed in the Appendix). They reveal that in the absence of NA inhibitors, WT-I223 has a definite replicative advantage over MUT-I223V in terms of the peak total viral load (1.5 × 10^10^ to 5.2 × 10^8^ RNA copies/mL, *p* = 0.002), infectious virus titer (5.4 × 10^7^ to 3.9 × 10^6^ PFU/mL, *p* < 0.001), and fraction of cells infected (0.96 to 0.04, *p* < 0.001). This is primarily caused by the increase in the eclipse phase (*τ*_*E*_) for the MUT-I223V which creates a significant delay in its viral replication cycle, enabling the WT-I223 to take the lead early and retain it; a clear fitness advantage.

Fig 2B presents a second simulated competition experiment between WT-I223 and MUT-I223V, but this time in the presence of oseltamivir. The standard, oseltamivir therapy regimen of 75 mg pill twice per day results in a minimum average plasma concentration of ∼ 350nM oseltamivir carboxylate [31–33]. Fig 2B presents a much more conservative scenario, with the inclusion of a simulated, oseltamivir concentration of 15nM, which corresponds to an efficacy of 97% and 85% against the WT-I223 and MUT-I223V strains, respectively (see Methods). Even with the relatively poor level of resistance offered by the I223V mutation, the presence of oseltamivir greatly reduces the fitness of the WT-I223 compared to that of the resistant MUT-I223V resulting in a lower peak of total virus (1.3 × 10^7^ to 1.9 × 10^9^ RNA copies/mL, *p* < 0.001), infectious virus (4.5 × 10^4^ to 1.3 × 10^7^ PFU/mL, *p* < 0.001), and a significant difference in the total fraction of cells infected (0.03 to 0.84, *p* < 0.001). If a higher dose of oseltamivir was administered, the relative fitness advantage of the MUT-I223V over the WT-I223 would be even more significant. In the presence of a NA inhibitor such as oseltamivir, the H1N1pdm09 MUT-I223V can overtake its oseltamivir-sensitive wild-type counterpart and become the dominant strain.

### Comparing the H275Y and I223V oseltamivir-resistant mutations

**Fig 3:**
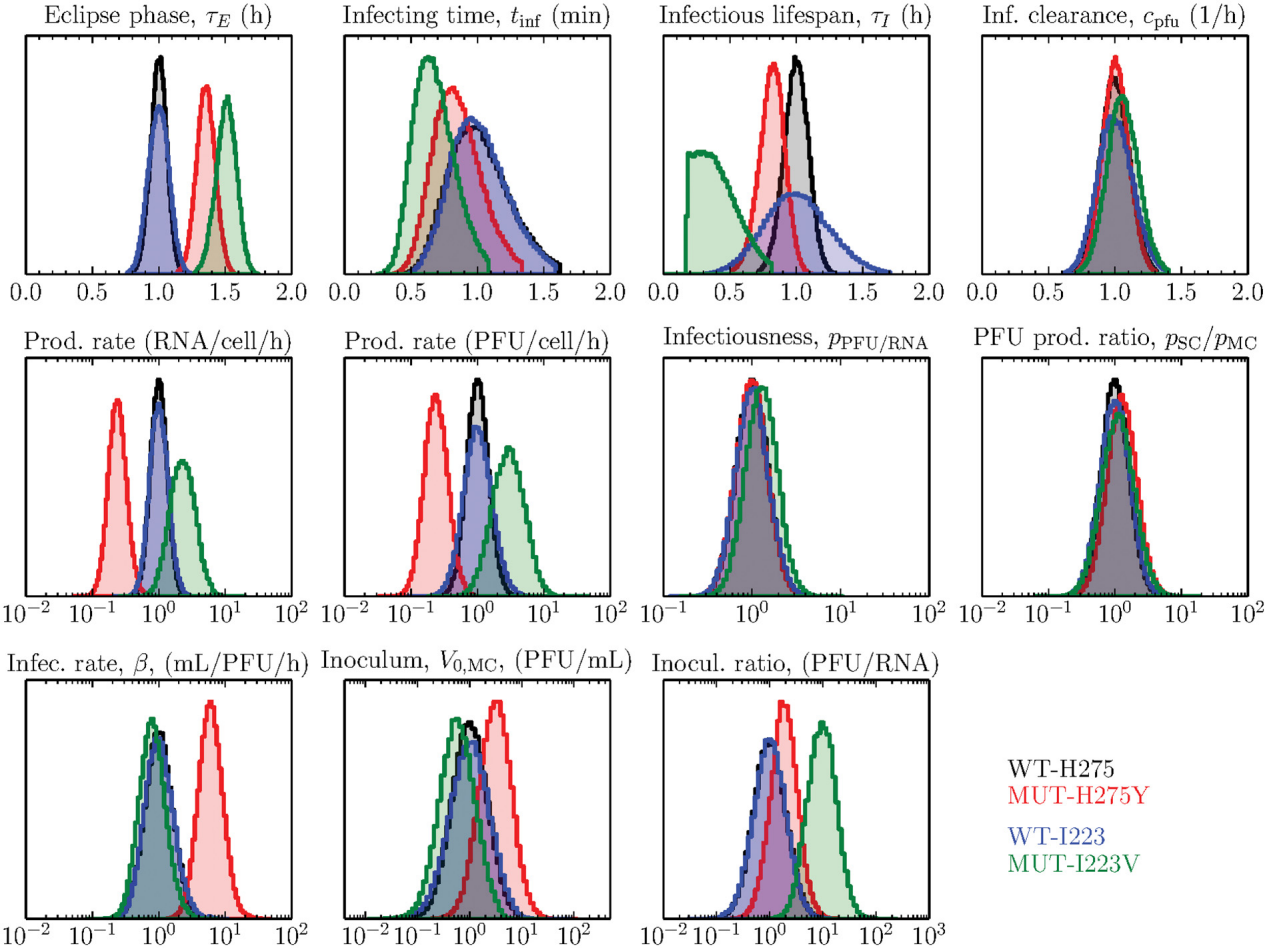
Impact of the H275Y and I223V NA mutations in the H1N1pdm09 background. The probability density functions of the key viral replication parameters characterizing the fitness of the WT-H275 and MUT-H275Y H1N1pdm09 strains (from earlier work, [18]), and that of the WT-I223 and MUT-I223V strains are presented side-by-side for comparison. To facilitate the comparison across the two separate experiments, the WT distributions have been centred at one (10^0^) and the H275Y and I223V mutants’ parameters are shown relative to their respective WT parameters.

In previous work we explored the effect of another N1 NA mutation conferring oseltamivir resistance, the H275Y mutation, using a different aliquot of the same A/Québec/144147/09 H1N1pdm09 strain sample [18]. Having two sets of assays, each with the wild-type and a single mutant, enables us to compare side-by-side the effects of the I223V mutation to those of the H275Y mutation in the same H1N1pdm09 background. In order to account for the inter-experimental variability discussed in the next section, we compare mutant strains between these two experiments in terms of relative parameter shifts from the wild-type strain in their respective experiment rather than in terms of their absolute parameter values. Specifically, since WT-I223 (current study) and WT-H275 (previous work) are different aliquots of the same strain sample, we express parameters for the MUT-I223V (or MUT-H275Y) in terms of fold-change from WT-I223 (or WT-H275). The results are summarized in Table 2 and illustrated in Fig 3.

**Table 2:** Comparative impact of the H275Y and I223V mutations*.

Parameter	MUT-H275Y/WT-H275		MUT-I223V/WT-I223	
	Median fold-change	Sign.	Median fold-change	Sign.
Eclipse period, *τ*_*E*_ (h)	1.35 [1.22–1.49]	***p* < 0.001**	1.51 [1.35–1.65]	***p* < 0.001**
Infecting time, *t*_infect_ (min)	0.84 [0.54–1.27]	*p* = 0.58	0.66 [0.42–1.04]	*p* = 0.20
Infectious lifespan, *τ*_*I*_ (h)	0.82 [0.61–1.00]	*p* = 0.17	0.39 [0.19–0.81]	***p* = 0.04**
Virion decay rate, *c*_PFU_ (h^−1^)	1.00 [0.81–1.21]	*p* = 0.99	1.06 [0.83–1.30]	*p* = 0.76
Total prod. rate, *p*_RNA_ (RNA/cell/h)	0.24 [0.13–0.45]	***p* < 0.001**	2.27 [0.98–5.3]	*p* = 0.13
Infect. prod. rate, *p*_PFU_ (PFU/cell/h)	0.24 [0.11–0.54]	***p* = 0.01**	2.90 [0.98–8.2]	*p* = 0.15
Virus infectiousness, *β* (mL/PFU/h)	6.0 [2.7–14.1]	***p* = 0.005**	0.80 [0.34–2.07]	*p* = 0.73
Inoculum infectiousness, $\frac{V_{\text{PFU}}(0)}{V_{\text{RNA}}(0)}$, $\left(\frac{\text{PFU}}{\text{RNA}}\right)$	1.9 [0.57–5.8]	*p* = 0.50	9.6 [2.8–32]	***p* = 0.02**
Multiplicity of infection (MOI)	19 [6.0–57]	***p* < 0.001**	0.47 [0.14–1.49]	*p* = 0.38
Prod. infectivity ratio, $\frac{p_{\text{PFU,SC}}}{p_{\text{PFU,MC}}}$	1.2 [0.5–3.5]	*p* = 0.75	1.1 [0.36–3.4]	*p* = 0.89

* Parameters are expressed as fold-changes in the parameters of the MUT strain relative to those of the WT strain in their respective experiment. Significance (Sign.) of differences between the MUT and its respective WT are provided as *p*-values.

Since both mutations are in the N1 NA and both cause resistance to oseltamivir, it is expected they will have a similar impact on the viral replication parameters. The two mutations are similar in the fact that both cause a significant (*p* < 0.001) increase in the length of the eclipse phase (*τ*_*E*_, 35% and 51% for H275Y and I223V, respectively), conferring a fitness disadvantage. They also cause a disadvantageous reduction in the length of the infectious lifespan (*τ*_*I*_), and an advantageous reduction in the time required for one infectious cell to infect one other (*t*_infect_), but only the reduction in *τ*_*I*_ for the I223V mutation was statistically significant (*p* = 0.04). Differences between the two mutations also emerge. As previously determined [18], the H275Y mutation is associated with a significant, disadvantageous reduction in total (RNA) and infectious (PFU) virus production rates (*p* < 0.001 and *p* = 0.01 respectively) which are over-compensated by an advantageous increase in the infectivity of the virus (*β*, 500%, *p* = 0.005). In contrast, the I223V mutation causes no such changes in virus production rates or virus infectivity. Overall, the H275Y mutation causes a mixture of disadvantageous (increase in eclipse phase length and a decrease in virus production) and advantageous (increase in virus infectivity, *β*) changes in viral replication parameters, leading to an overall fitness similar to that of its WT counterpart, as reported previously [18]. In contrast, the I223V mutation appears to cause only disadvantageous changes (increase in the eclipse phase length and a decrease in the infectious cell lifespan or duration of virus production) with no significant compensatory advantages, suggesting it is likely overall less fit than its wild-type counterpart.

**Fig 4:**
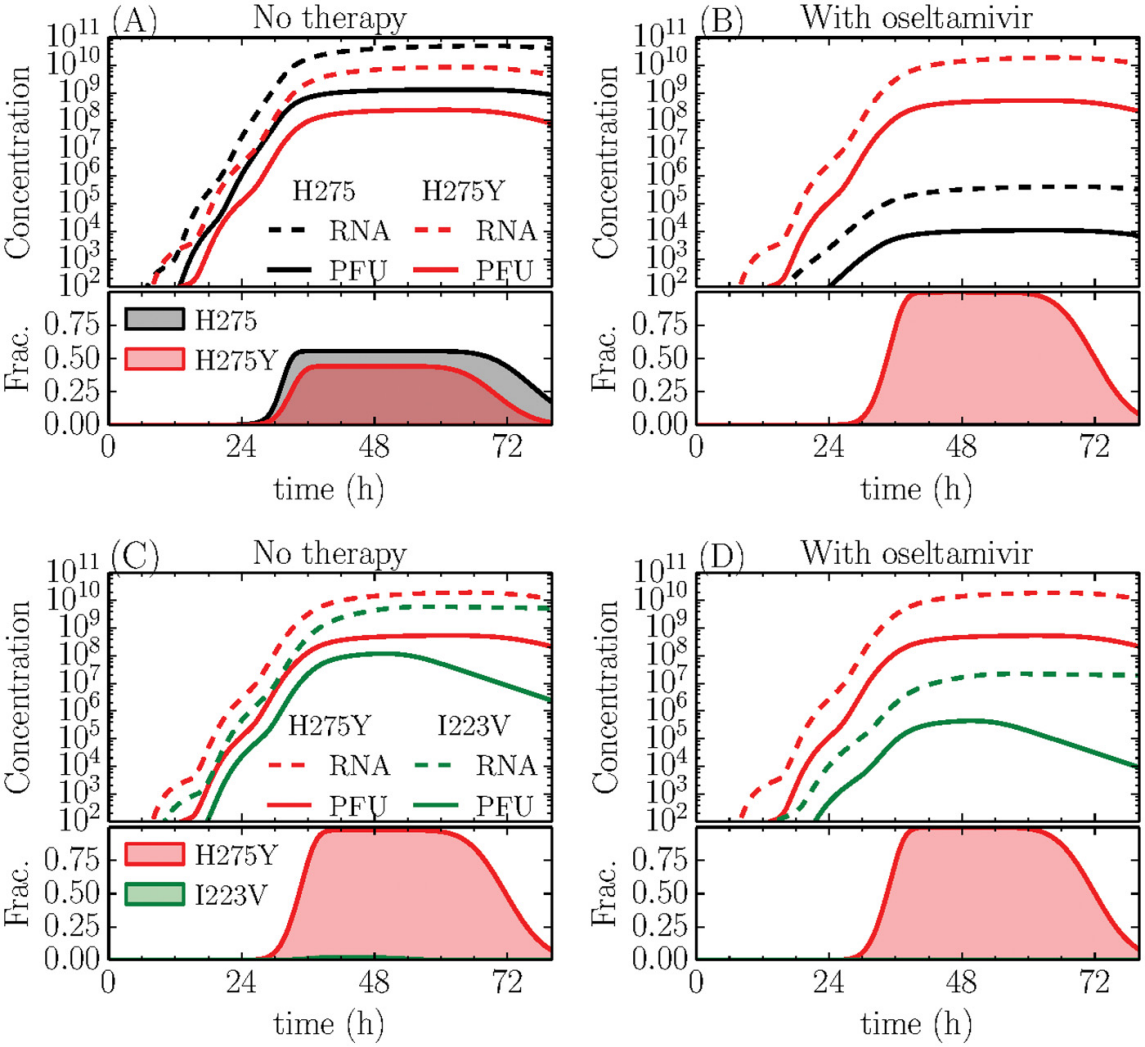
Simulated competition between the wild-type, MUT-H275Y and MUT-I223V strains. The infectious (PFU/mL) and total (RNA/mL) viral load (top), and the fraction of infectious cells infected by each strain (bottom) are shown. (A) Without therapy, the fitness of the WT and MUT-H275Y strains is comparable. (B) In the presence of oseltamivir, the WT has a replicative disadvantage over the MUT-H275Y. (C) Without therapy, the MUT-H275Y strain already has a fitness advantage over the MUT-I223V strain. (D) In the presence of oseltamivir, this advantage is increased.

Fig 4 shows the fitness of each strain relative to another in terms of peak total (RNA) and infectious (PFU) virus concentration and fraction of cells infected achieved in both the absence and presence of oseltamivir. Quantitative details of these three outcomes and statistical significance are available in the Appendix. For the simulated competition between the WT-H275 vs MUT-H275Y (Fig 4A), the wild-type produces significantly more total virus (RNA copies, *p* = 0.04), but comparable concentrations infectious virus, infecting an equivalent fraction of the cells. In the presence of oseltamivir (Fig 4B), the wild-type’s virus production rates are highly-suppressed, and MUT-H275Y gains a significant competitive advantage producing more total and infectious virus and infecting the most cells.

Comparing the MUT-H275Y with the MUT-I223V reveals that in the absence of oseltamivir (Fig 4C) the MUT-H275Y does produce more infectious virus (*p* = 0.04) and infects more cells (*p* < 0.001), but produces a similar amount of total virus compared with the MUT-I223V. Here, both MUT-H275Y and MUT-I223V strains have similar eclipse phase lengths, but the MUT-H275Y has a significantly increased viral infectiousness *β*, relative to the MUT-I223V strain. In the presence of oseltamivir (Fig 4D), the fitness advantage of the MUT-H275Y is emphasized, dominating the MUT-I223V strain significantly on all fronts, owing to its higher resistance to oseltamivir compared to the MUT-I223V strain. Higher doses of oseltamivir will further increase this fitness advantage. This is in accordance with the frequency of emergence of MUT-H275Y in patients undergoing oseltamivir therapy [9–12], as ∼ 350nM oseltamivir corresponds to an efficacy of 99.9% for the wild-type, 99.3% for the MUT-I223V but only 44% for the MUT-H275Y.

### Validation, reproducibility and inter-experimental variability

In previous work we determined the viral replication parameters characterizing the fitness of the wild-type A/Québec/144147/09 H1N1pdm09 strain (WT-H275) and compared them against those of its oseltamivir-resistant variant containing the single mutation H275Y in its NA (MUT-H275Y) [18]. Here we are evaluating a different aliquot of this same A/Québec/144147/09 H1N1pdm09 wild-type strain sample (WT-I223) against another oseltamivir-resistant variant containing, instead, the single mutation I223V in its NA (MUT-I223V). Hence, if our method to extract viral replication parameters is robust, and if viral replication parameters are not significantly dependent on variability in conditions between experiments, then we would expect the parameters extracted in our previous work for the wild-type strain (WT-H275) to match those extracted here for a different aliquot of the same strain sample (WT-I223). However, in a comparison of the parameter estimates for the two WT aliquots, we find that all virus replication parameter estimates, except for the length of the eclipse phase, are significantly different (Table 3). This suggests that the parameter estimates extracted by our approach (experimental assays plus mathematical modelling analyses) vary significantly across separate experiments for a given strain.

**Table 3:** Inter-experimental changes in the viral kinetic parameters for WT H1N1pdm09*.

Parameter	WT-I223 (current) Median [95% CI]	WT-H275 (previous) Median [95% CI]	(I223 vs. H275) Significance
Eclipse period, *τ*_*E*_ (h)	6.9 [5.9–8.0]	6.9 [6.0–7.7]	*p* = 0.98
Infecting time, *t*_infect_ (min)	19.5 [12.5–29.5]	8.4 [5.3–12.9]	***p* = 0.007**
Infectious lifespan, *τ*_*I*_ (h)	28 [16–43]	46 [37–54]	***p* = 0.03**
Virion decay rate, *c*_PFU_ (h^−1^)	0.093 [0.070–0.119]	0.145 [0.116–0.176]	***p* = 0.01**
Total prod. rate, *p*_RNA_, (RNA/cell/h)	560 [300–1060]	7460 [4240–13100]	***p* < 0.001**
Infectious prod. rate, *p*_PFU_ (PFU/cell/h)	6.0 [2.3–16.2]	350 [170–750]	***p* < 0.001**
Virus infectiousness, *β* (mL/PFU/h)	3.1 × 10^−6^[1.2–8.7]	3.0 × 10^−7^[1.2–7.7]	***p* < 0.001**
Inoculum infectiousness, $\frac{V_{\text{PFU}}(0)}{V_{\text{RNA}}(0)}$, $\left(\frac{\text{PFU}}{\text{RNA}}\right)$	3.8 × 10^−6^[0.9–14.3]	2.7 × 10^−7^[0.7–11]	***p* = 0.008**
Multiplicity of infection (MOI)	8.4 × 10^−7^ [2.3–29.3]	4.2 × 10^−8^ [1.3–13.7]	***p* < 0.001**
Prod. infectivity ratio, $\frac{p_{\text{PFU,SC}}}{p_{\text{PFU,MC}}}$	3.4 × 10^−3^ [1.2–9.4]	2.4 × 10^−4^ [0.9–6.0]	***p* < 0.001**

* Median and 95% confidence intervals for MCMC parameter values from a previously published assay set (WT-H275) and the current analysis (WT-I223), a separate aliquot of the same A/Québec/144147/09 H1N1pdm09 strain sample. Significance of differences are provided as *p*-values.

**Fig 5:**
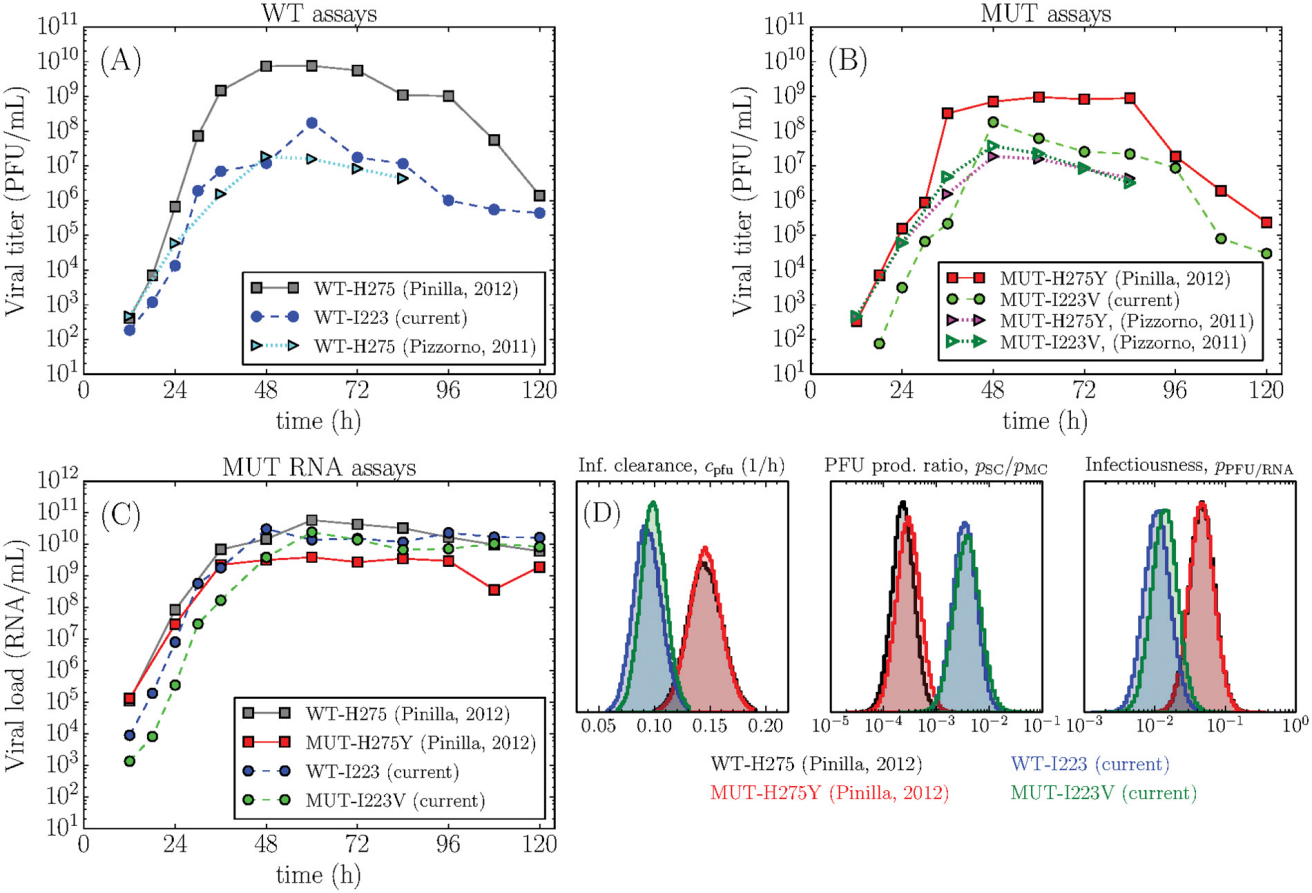
Differences between inter-experimental replicates. (A) Infections with the same H1N1pdm09 wild-type strain illustrate that viral titer growth rates, peak values, and decay rates can vary noticeably between three experiments performed on different dates. (B) Infections with either the MUT-H275Y or the MUT-I223V single mutants over two separate experiments. The peak viral titers are more similar to the wild-type performed in the same experiment (comparing to left graph), than within the same strain in different experiments. (C) Recorded RNA titers either from the current study or previously presented (Pinilla, 2012 in [18], Pizzorno, 2011 in [34]) and reproduced here for comparative purposes. (D) Probability density functions of parameters for the WT and mutant pair of strains in either the current (blue, green) or previous (black, red) experiment illustrate that some infection parameters are clearly more experiment-specific than strain-specific, pointing to a genuine impact of inter-experimental variability on the actual viral replication parameters.

To investigate the origin of these inter-experiment differences in parameter estimates, we consider the time-course viral loads for three separate MC infection experiments with the wild-type A/Québec/144147/09 H1N1pdm09 strain, conducted using different aliquots of the same sample, in the same laboratory, by different personnel, following the same procedure (Fig 5A–5C). We can see that various features of the time-course data sets differ significantly between experiments for the same strain. For example, the rate of infectious (PFU) virus decay exhibited by WT-H275 (Pinilla 2012, [18]) is greater (more rapid) than that seen with WT-I223 (current study). Since the experimental infectious virus decay rate is equal to the model’s virus clearance rate parameter (*c*_PFU_), it is not surprising that *c*_PFU_ was found to be larger for WT-H275 than WT-I223. In other words, the significant difference in this parameter is due to a genuine difference in the experimental infectious virus decay rates between the two experiments that the mathematical analysis enables us to quantify. Experimentally, a decrease in the rate at which virions lose infectivity, i.e. improved virus stability, could be attributable to variables such as colder overall experimental temperatures (e.g., from sharing an incubator with another experiment) or use of a different stock of medium.

Another stark difference is the peak infectious viral titer (PFU) observed for WT-H275 (Pinilla 2012, [18]), which is significantly higher than that seen for WT-I223 (current study) and for one other distinct experiment using the same wild-type strain (Pizzorno 2011, [34]). The model equations imply that the peak virus value is given by *V*_peak,PFU_ = *I* ⋅ *p*_PFU_/*c*_PFU_. At the time of peak, approximately all of the cells are infected (*I* = 1), so, given the modest change in *c*_PFU_ described above, the higher viral titer peak observed experimentally implies a larger value of the virus production rate parameter, *p*_PFU_, for WT-H275 than for WT-I223. Again, we see that the significant difference in extracted parameter values is associated with a clearly visible difference in the experimental data. If the viral production rate per cell is genuinely changing between experiments, it could be due to variations in sialic acid expression on the cell culture. Our experiments are conducted on MDCK cells that are transfected to express *α*-2,6 sialic acid receptors in greater amounts—making their expression level more consistent with that on the surface of epithelial cells in the human upper respiratory tract, and thus improving the affinity of human influenza virus strains for these cells [35, 36]. A decreased expression of these receptors in the cells used for the WT-H275 experiment, compared to those infected with WT-I223, could increase both virus production (larger *p*_PFU_) and virus release rates (shorter infecting time, *t*_infect_), consistent with the shifts seen in these parameters in Table 3.

While inter-experimental variability could be attributable to (1) stochasticity of the data or (2) systematic bias due to the variation of “hidden” variables which have not been measured in the laboratory or accounted for in the modelling, it seems clear from the data presented here that the latter is the true cause. In previous work, we estimated parameter values using our model, and then used these estimates to simulate and successfully predict the course and outcome of a true competition experiment conducted experimentally between the WT-H275 and MUT-H275Y strains [18]. This evidence—along with the fact that the three-replicate averages of each time-course data set follow clear trends with little noise—suggests that our analysis method is robust and successfully extracts the true experimental parameters characterizing the viral replication kinetics observed within a single set of experiments. But, given the changes we highlight here in the experimentally-observed viral kinetics and in the corresponding viral replication parameters identified by our analysis, it is clear that these measures are sensitive to minor changes in experimental conditions.

Given the evident inter-experimental variability, it remains a question whether any results can be compared between experiments. While the results presented in Table 3 demonstrate that specific parameter values certainly cannot be compared, there is some evidence that the changes in experimental conditions leading to this variability affect virus strains in the same way. Evidence of this is presented in Fig 5D, which shows that certain parameters are clearly experiment-specific, rather than strain-specific. Those parameters that were found to be the same for the WT and MUT strains, were found to be the same in each of the two experiments. The extracted parameter value changed significantly between experiments, but in each case the value was identical for the two tested strains. It is a reasonable course of action, therefore, to compare the parameter values of the two mutant strains by evaluating their fold-change difference relative to the identical WT strain.

## Discussion

In this work we presented a set of experiments investigating the impact of the introduction of a single isoleucine-to-valine mutation at residue 223 of the N1 neuraminidase (NA) protein of the A/Québec/144147/09 H1N1pdm09 strain. These experiments were performed on MDCK-2,6 (SIAT-1) cells in the absence of NA inhibitors, and both total (RNA) and infectious (PFU) virus concentrations were measured. Experimentally, we found that the MUT-I223V strain reached comparable total and infectious viral titers to those seen with the wild-type (WT), but the viral kinetics observed for the MUT-I223V strain was delayed by a few hours compared to that of its WT counterpart. Mathematical analyses of the experimental data identified that the I223V mutation in the H1N1pdm09 background causes a significant increase in the length of the eclipse phase (*τ*_*E*_, from 6.9h to 11h, *p* < 0.001), and a significant decrease in the duration of virus production by infected cells (or infectious lifespan, *τ*_*I*_, from 28h to 11h, *p* = 0.04), both disadvantageous to the fitness of the MUT-I223V strain relative to the WT. These findings explain the observed delay to peak viral titer for the I223 mutants [23], and are similar to those we previously reported for the changes caused by a histidine-to-tyrosine mutation at residue 275 of the N1 NA protein of the same strain [18]. In the MUT-H275Y strain, the significant increase in the eclipse phase length (from 6.9h to 9.4h, *p* < 0.001) was accompanied by a decrease in the total viral production rate (from 7460 to 1770, *p* < 0.001) compensated by an increase in the infectiousness of the virions produced. This more advantageous change was not observed in the MUT-I223V strain. Both the I223V and H275Y mutations were introduced into the H1N1pdm09 background via reverse genetics such that the MUT-H275Y and MUT-I223V strains differ from the WT strain only in their single, respective mutation. As such, we can be certain that the effects observed are the result of the mutation of interest, and not of other mutations elsewhere.

Competition experiments were simulated to evaluate the overall fitness of one strain relative to another. In these experiments, two strains inoculated in equal quantities fight over a finite number of available susceptible cells. Evaluating whether the MUT strain infects more cells or produces higher virus concentrations than the WT strain provides insight into the potential of a virus population carrying this mutation to develop and grow within one individual or to transmit between individuals. The simulated competition experiment between the WT and MUT-I223V strains in the absence of oseltamivir shows that the total viral load (1.5 × 10^10^ to 5.2 × 10^8^ RNA copies/mL, *p* = 0.002), the infectious viral titer (5.4 × 10^7^ to 3.9 × 10^6^ PFU/mL, *p* < 0.001) and the fraction of cells infected (0.96 to 0.04, *p* < 0.001) would be dominated by the WT strain, whereas the MUT-H275Y strain somewhat matched the fitness of the WT strain infecting a comparable fraction of cells and producing a comparable amount of infectious virus. Our mathematical approach also allowed us to simulate a competition experiment between the MUT-H275Y and MUT-I223V strains. We found that these two strains produce similar amounts of total virus (RNA) when in direct competition, but that the MUT-H275Y strain would produce significantly more infectious virus (*p* = 0.04) and infect a larger fraction of cells (*p* < 0.001). In the presence of oseltamivir, the relative fitness of either single mutant dominates over the wild-type. A simulated competition experiment between WT-I223 and MUT-I223V shows a larger total (1.3 × 10^7^ to 1.9 × 10^9^ RNA copies/mL, *p* < 0.001) and infectious (4.5 × 10^4^ to 1.3 × 10^7^ PFU/mL, *p* < 0.001) viral titer, and fraction of infected cells (0.03 to 0.84, *p* < 0.001) for the MUT-I223V strain in the presence of 15nM oseltamivir. This replicative advantage will further increase at higher concentrations and supports the observations of the emergence of mutations at residue I223 in patients undergoing oseltamivir therapy [20, 21].

The WT strain used in this study was identical to strain A/Québec/144147/09 H1N1pdm09 analyzed in our previous work using the same protocols but different lots of cells, reagents, and media [18]. This provided us with a unique opportunity to evaluate the reproducibility of our observed experimental viral kinetics and of the parameters extracted by our analysis across experiments. A comparison of the parameters extracted by our method for the WT strain in the present experiments to those from our previous work revealed that almost all viral replication parameters had changed significantly, with the exception of the eclipse phase. We showed that the changes in the extracted parameters are genuine (correctly extracted by our analysis) and can be directly related to visible differences in the experimentally-observed virus kinetics exhibited by the strain in the two separate experiments. These changes are attributable to inter-experimental variability.

When comparing data across separate experiments performed days or even months apart, one expects significant variability or “batch effects” due to differences in the stock of cells, reagents or media used, personnel conducting the experiments, the overall experimental conditions such as atmospheric pressure, relative humidity, or temperature, an effect that is well-known and recognized [26]. It is possible to control for these variations by, for example, having the same person run all arms of the study at the same time, performing the same experiments, with the same lot of cells, reagents, and media. In this work, the two sets of triplicates for the WT and MUT-I223V were all performed exactly in this manner (two sets of plates processed in the same manner, at the same time, by the same individual) so as to virtually eliminate this variability. However, to improve the likelihood that findings will hold true, the key results of an experiment should be ***reproducible***, i.e. hold true despite reasonable inter-experimental variations (the investigators repeating the experiments, the lots of cells and media used), rather than being merely ***replicable***, i.e. hold true only in the near absence of experimental variations [27–30]. This will be the case if the sources of inter-experimental variability (e.g., temperature, precise content of bovine serum) affect all viral strains equivalently within one experiment. Then, the properties of one strain relative to another (e.g., strain A has a greater virus production rate than strain B) will be preserved between separate experiments, even if their actual, absolute parameter values change significantly. Experimentally, this would mean that, for example, if the peak viral titer of the WT is significantly above that of the MUT in one experiment, then this should remain true in any other experiment as well, irrespective of personnel or stock of experimental consumables. Thus, if inter-experimental variations affect all strains equivalently, then the fitness of one strain relative to another (e.g., strain A produces more virus than strain B) will be robust in the face of inter-experimental variability, and the results reported herein, in our previous work [18], and in the works of countless others using a similar infection system will hold and be robust.

Herein (Fig 5D), we have shown that for some parameters, the inter-experimental variability does indeed affect both strains similarly (e.g., the rate at which infectious virus loses infectivity). This suggests that statistically significant relative changes in the parameters between the WT and its corresponding MUT strain (e.g., the WT produces 10 × more virus than the MUT)—rather than the absolute value of the parameters—may be preserved across experiments. With increasing concerns regarding the lack of reproducibility in experimental health research, mathematical modelling offers an elegant and much needed solution to quantify, identify, and even negate inter-experimental variability, and provide robust, reproducible results.

Together, these findings suggest that future work to quantitatively compare multiple virus strains (1) can utilize a reference strain across multiple experiments to facilitate a meaningful fold-change analysis of parameter values, despite inter-experimental variability; and (2) should incorporate multiple distinct replicates—experiments which are performed some time apart, with different materials (cells, reagents, media)—in order to accurately determine viral-kinetics parameter values and the corresponding uncertainty in those measures, taking into account minor variability of experimental conditions. The mathematical modelling framework used herein offers an ideal platform with which to quantify, evaluate and account for inter-experimental variability, and to detect experimental issues that can negatively impact the significance of the findings.

## Methods

### Viruses

Recombinant H1N1pdm09 WT-I223 (wild-type) and MUT-I223V (mutant) viruses were rescued by reverse genetics from the first sample of 2009 pandemic influenza A (H1N1pdm09) isolate in Québec, Canada (A/Québec/144147/09) as described previously [18, 34]. The WT-I223 and MUT-I223V differ only by a single isoleucine-to-valine amino acid substitution at residue 223 of the N1 NA protein. The WT-I223 is identical to the H1N1pdm09 WT-H275 strain previously analyzed [18], and the MUT-H275Y differs from the wild-type by only a single histidine-to-tyrosine substitution at residue 275.

### In vitro replication experiments

We performed single-cycle (SC), multiple-cycle (MC), and mock-yield (MY) assays on both the H1N1pdm09 strain A/Québec/144147/09 (WT-I223) and its mutant counterpart (MUT-I223V). These viral yield experiments were conducted on Madin-Darby canine kidney (MDCK) cells modified to express *α*-2,6 sialic acid receptors (MDCK*α*2,6) at levels more consistent with those found on the surface of epithelial cells in the human upper respiratory tract, as in the previous experiment that examined the same H1N1pdm09 wild-type (WT-H275) and the MUT-H275Y single mutant [18]. Cells were grown in a maintenance medium composed of DMEM with added FBS and GVF (gentamycin, vancomycin, fungizone) antibiotic solution.

For the SC assay performed at a high multiplicity of infection (MOI), confluent cells grown in 12-well plates (containing 10^6^ cells per well) were infected by replacing the medium with 0.5mL of infection medium (which consists of a maintenance medium without FBS, but containing 1 µg/µL of TCPK) with a viral inoculum at an MOI of 4 (4 × 10^6^ PFU). The well plates were then incubated for one hour at 37°C in 5% CO_2_. After incubation, the supernatant was removed, cells were quickly washed once with acidic saline solution (0.9% NaCl in water, pH 2.2) and twice with phosphate-buffered saline (PBS, pH 7.4), fresh infection medium (1mL) was added to each well, and the plates were returned to the incubator. Subsequently, the supernatant liquid was collected in triplicate at each time point, one sample each from three separate wells, terminating the experiment for those specific wells. Three new wells per strain are thus harvested every hour from 2 hours post-infection (hpi) until 10hpi, and then at 12hpi, 15hpi and 18hpi.

In the MC assay, cells were grown to confluence in 12-well plates. The maintenance medium was then replaced with 0.5mL of infection medium containing a viral inoculum at an MOI of 5 × 10^−5^ (50 PFU). After a 1-hour incubation period at 37°C in 5% CO_2_, the medium was replaced (no rinsing) with 1.0mL of fresh infection medium (with no virus). The plates were returned to the incubator and supernatant harvested in triplicate every 6h, from 12h postinfection (hpi) until 36hpi, and then every 12h thereafter until 120hpi. For the MY assay, designed to measure the infectious viral decay rate, known titers of the virus were incubated in 12-well plates with the same infection medium, but without cells. These samples were harvested in triplicate at 0h, 24h, 48h, and 72h and frozen for later titration.

### Infectious and total virus quantification

All supernatant samples were harvested in triplicate and frozen at -80°C until their use for either RNA isolation or viral titration by standard plaque assay on MDCK*α*2,6 (SIAT-1) cells. The infectious virus concentration in the supernatant (*V*_PFU_, in PFU/mL) was recovered from all timepoints in all three experiments (SC, MC, and MY), and the total virion concentration in the supernatant (*V*_RNA_, in RNA counts/mL) was also measured at all timepoints, but only in the SC and MC assays. The infectious virus concentration was measured by infecting confluent MDCK*α*2,6 cells by using serial dilutions (in factors of ten) of the viral samples in infection medium. After one hour of incubation at 37°C in 5% CO_2_, the supernatant was removed and infected cells were overlaid with DMEM 2x/1.6% agarose (v/v) containing 1 µg/µL of TPCK. After a 72h incubation period the agarose was removed, cells stained and plaques counted to calculate the number of plaque-forming units (PFU) of each sample.

RNA extraction was performed on 200 µL of each supernatant sample with the MagNA Pure LC total nucleic acid isolation kit (Roche Applied Science, Mannheim, Germany), according to manufacturer’s instructions on the robotic MagNA Pure instrument. The discriminatory one-step real-time PCR conditions were conducted in 25 µL volumes containing 6.25 µL of Taqman Fast Virus 1-Step Master Mix (Applied Biosystems, Foster city, Canada), 0.8 µL of both the reverse and the forward primers, 2.0 µL of each probe and 4.0 µL of RNA extract. The following thermal profile was used: a single cycle of reverse transcription for 30min at 60°C, 5min at 95°C for reverse transcriptase inactivation and DNA polymerase activation followed by 45 amplification cycles of 20 s at 95°C and 1min at 63°C each (annealing-extension step). Thresholds were set manually for analysis. Data acquisition was performed in both FAM and CY-5 filters during the annealing/extension step in a Light Cycler 480 real-time thermocycler (Roche, Germany). One reaction using both probes was performed for each sample and each PCR reaction was completed in triplicate. Primers were designed using Primer Express 2.0 software (Applied Biosystems, Foster City, CA, USA) with melting temperatures (Tm) of 58–60°C. We used A/H1N1 specific NA primers with forward primer 5’-GGG CAG TGG CTG TGT TAA AG-3’ and reverse primer 5’-TTG GTC CAT CGG TCA TTA CA-3’ to generate a 138bp amplicon. The specific probes for the I223V discrimination assay were designed with Exiquon LNA Oligo design tools and follow the guideline for design of LNA probes that improve mismatch discrimination [37]. The specific WT-I223 probe (5’-ACA A **T** **A** **T**A **T** **T**G **A** GAA C6-FAM/BHQ-1) and MUT-I223V probe (5’-ACA A **TG** **T**A **T** **T**G **A** GAA C-Cy5/BHQ-2) were designed around nucleotide position 667 of the NA gene. Six LNA bases were incorporated in the probes (bold, underlined) allowing them to be shorter with increased discriminative capacities.

### Mathematical model

The SC and MC infection experiments were simulated numerically using a multi-compartment ordinary differential equation (ODE) model, introduced and described previously [18]. The ODE model (Eq (1)) considers both the infectious (*V*_PFU_, measured in PFU/mL) and total (*V*_RNA_, measured in RNA counts/mL) virus concentration released by infected cells into the supernatant over the course of the infection.

$$\begin{array}{ccc} \frac{dT}{dt} & = & -\beta TV_{\text{PFU}}\\ \frac{dE_{1}}{dt} & = & \beta TV_{\text{PFU}}-\frac{n_{E}}{\tau_{E}}E_{1}\\ \frac{dE_{i}}{dt} & = & \frac{n_{E}}{\tau_{E}}E_{i-1}-\frac{n_{E}}{\tau_{E}}E_{i}\;\;\;\text{for}\;i=\left(2,\ldots ,n_{E}\right)\\ \frac{dI_{1}}{dt} & = & \frac{n_{E}}{\tau_{E}}E_{n_{E}}-\frac{n_{I}}{\tau_{I}}I_{1}\\ \frac{dI_{j}}{dt} & = & \frac{n_{I}}{\tau_{I}}I_{j-1}-\frac{n_{I}}{\tau_{I}}I_{j}\;\;\;\text{for}\;j=\left(2,\ldots ,n_{I}\right)\\ \frac{dV_{\text{PFU}}}{dt} & = & p_{\text{PFU}}\sum_{j=1}^{n_{I}}I_{j}-c_{\text{PFU}}V_{\text{PFU}}\\ \frac{dV_{\text{RNA}}}{dt} & = & p_{\text{RNA}}\sum_{j=1}^{n_{I}}I_{j}-c_{\text{RNA}}V_{\text{RNA}} \end{array}$$ (1)

Fractional cell population is tracked as it progresses through an uninfected or target phase (*T*), several eclipse (*E*_*i*_, *i* = 1…*n*_*E*_) and infectious (*I*_*j*_, *j* = 1…*n*_*I*_) compartments before reaching cell death and ceasing virus production. The eclipse and infectious phases have an average duration of *τ*_*E*_ and *τ*_*I*_, respectively. The eclipse phase (*E*_*i*_) corresponds to the period between successful infection of the cell by the influenza virus and the release of the new infectious virus by this same newly-infected cell. After the eclipse phase cells enter the infectious phase, throughout which the production rates of infectious and total virus are considered constant. The multiple compartments making up the eclipse and infectious phases mean that the time spent by cells in each of these compartments follows an Erlang distribution, which we have shown in past work to be biologically appropriate [18, 38]. This model is identical to the one introduced and used in [18], with the single exception being the inclusion of a dedicated parameter, *p*_*RNA*_, describing the production rate of total viral RNA by the infected cells. This change was made to reflect the inclusion of measurements of total extracellular influenza virion concentration in the supernatant, model variable *V*_RNA_, in both SC and MC assays, whereas in previous work this quantity was only measured in the MC assay [18]. The production rates of infectious and total virus per cell reported herein are obtained by multiplying model parameters *p*_PFU_ (PFU/mL per hour) and *p*_RNA_ (RNA copies/mL per hour) by the volume of supernatant in each culture well (1.0mL) and dividing it by the number of cells per well (10^6^ cells). The infecting time, *t*_infect_, represents the time elapsed between the production of the first viral progeny from a single infected cell and the eclipse (latent) infection of another cell by that progeny within a completely susceptible cell population [18]. It is given by

$$\begin{array}{c} t_{\text{infect}}=\sqrt{\frac{2}{p_{\text{PFU}}\beta }} \end{array}$$

The python function scipy.integrate.odeint was used to solve the model in Eq (1), and the python module emcee was used to perform the Markov chain Monte Carlo (MCMC) to estimate the parameters’ probability density function [39, 40]. Complete details of the method we followed to extract the virus replication parameter distributions can be found in the Appendix.

### Mathematical simulation of strain competition

In modelling competition between a pair of strains (denoted here as strains 1 and 2), a modified version of the model in Eq (1) was used wherein the equation for the susceptible, target cell fraction was replaced by

$$\begin{array}{c} \frac{dT}{dt}=-\beta_{1}TV_{\text{PFU,1}}-\beta_{2}TV_{\text{PFU,2}} \end{array}$$

where *β*_1_ (or *β*_2_) and *V*_PFU,1_ (or *V*_PFU,2_) are the infectivity and infectious virus concentration of virus strain 1 (or 2), respectively. All other equations are as in Eq (1), but with two sets of equations for *E*_*i*_, *I*_*j*_, *V*_PFU_, and *V*_RNA_, one set for each strain. Our modelling approach assumes that co-infection is not possible, namely when a cell is infected by one strain it cannot be co-infected by the other. Complete details of the parameters and method used to simulate the competition experiments are presented in the Appendix.

### Mathematical simulation of antiviral therapy

Neuraminidase inhibitors (NAIs) such as oseltamivir and peramivir suppress influenza infections by inhibiting the release of newly formed virus buds from the surface of infected cells. Since virus release is not explicitly represented in mathematical models of influenza infections, previous works chose to represent the effect of NAIs as a reduction in the rate of virus production [41, 42]. In our model (Eq (1)), this is achieved by modifying only the virus concentration differential equations, namely

$$\begin{array}{ccc} \frac{dV_{\text{PFU}}}{dt} & = & \left(1-\varepsilon \right)\;p_{\text{PFU}}\sum_{j=1}^{n_{I}}I_{j}-c_{\text{PFU}}V_{\text{PFU}}\\ \frac{dV_{\text{RNA}}}{dt} & = & \left(1-\varepsilon \right)\;p_{\text{RNA}}\sum_{j=1}^{n_{I}}I_{j}-c_{\text{RNA}}V_{\text{RNA}} \end{array}$$

where

$$\begin{array}{c} \varepsilon =\frac{\varepsilon_{\text{max}}\cdot \text{Dose}}{{\text{IC}}_{50}+\text{Dose}} \end{array}$$ (2)

and Dose is the NAI dose concentration used, *ɛ*_max_ is the maximum efficacy of the antiviral in blocking the virus production rate, and IC_50_ (50% inhibitory concentration) is the antiviral Dose at which the efficacy is half its maximum (i.e., when Dose = IC_50_, *ɛ* = *ɛ*_max_/2). In simulating therapy, we assume that the NAI dose concentration is constant throughout the duration of the experimental infection (*ɛ* = constant) and set *ɛ*_max_ = 1. Previous work has measured a 6-fold increase in oseltamivir resistance for the MUT-I223V single-mutant (IC_50_ = 0.46nM vs. 2.63nM) [13], and a 980-fold increase for the MUT-H275Y (IC_50_ = 451.9nM). Table 4 shows the measured IC_50_ for the strains in question and their corresponding efficacies as per Eq (2) for selected concentrations of oseltamivir.

**Table 4:** Calculated oseltamivir efficacies as a function of dose*.

OST Dose	Strain (IC_50_)	WT-H275 (0.46 nM)	MUT-H275Y (451.9 nM)	MUT-I223V (2.63 nM)
15 nM	*ɛ* =	0.97	0.03	0.85
50 nM	*ɛ* =	0.99	0.10	0.95
350 nM	*ɛ* =	0.999	0.44	0.993

* IC_50_ values are from previously-published work [13] and antiviral efficacies (*ɛ*) at various oseltamivir (OST) doses are computed using Eq (2).

## Appendix

### Extraction of virus replication parameter distributions

In order to simulate the MY experiment, a simple linear regression was employed, namely

$$\begin{array}{c} \ln\left[V_{\text{PFU}}\left(t\right)\right]=\ln\left[V_{\text{PFU}}\left(0\right)\right]-c_{\text{PFU}}\;t \end{array}$$ (3)

where *c*_PFU_ is the decay rate of virus infectivity as in Eq (1), and ln[*V*_PFU_(0)] is chosen so as to minimize the sum of squared residuals between the data and the MY model described in Eq (3). Therefore,

$$\begin{array}{c} \ln\left[V_{\text{PFU}}\left(0\right)\right]=\frac{1}{N_{\text{pts}}}\sum_{i=1}^{N_{\text{pts}}}(c_{\text{PFU}}\;t_{i}+\ln\left[V_{\text{PFU}}\left(t_{i}\right)\right]) \end{array}$$

where *N*_pts_ is the total number of data points collected for this strain in the MY experiment, and {*t*_*i*_, *V*_PFU_(*t*_*i*_)} are the collection time and the infectious virus concentration (PFU/mL) for the *i* ^th^ data point.

Our model, presented in Eq (1), has a total of 9 parameters (*β*, *τ*_*E*_, *n*_*E*_, *τ*_*I*_, *n*_*I*_, *p*_PFU_, *c*_PFU_, *p*_RNA_, *c*_RNA_) and 2 unknown initial conditions (*V*_PFU_(0) and *V*_RNA_(0)). For a given set of kinetic parameters and initial conditions, we use Eqs (1) and (3) to simultaneously simulate the MC, SC and MY experiments using identical parameters across all three assay types, with some exceptions mentioned below. The goodness of a particular fit (i.e., of a particular parameters set) was measured as the sum of the squared residuals (SSR) between the 5 curves predicted by the model (*V*_PFU_ for MC, SC, MY and *V*_RNA_ for MC and SC) and their corresponding experimental virus concentration measurements, all equally weighted. Since measurements were made in triplicate at each time point, the three corresponding squared residuals were summed at each time point.

The SC and MC infection assays are modelled with all cells initialized to the uninfected or target state *T* at the start of the infection, *t*_start_, namely *T*(*t*_start_) = 1 and *E*_*i*_(*t*_start_) = *I*_*j*_(*t*_start_) = 0 for all *i* and *j*. In previous work [43], we have shown that

$$\begin{array}{c} \text{MOI}=\frac{\beta V_{\text{PFU}}\left(0\right)}{c_{\text{PFU}}}[1-\exp(-c_{\text{PFU}}\cdot t_{\text{incub}})]\; \end{array}$$ (4)

where MOI is the multiplicity of infection obtained after a *t*_incub_ incubation period with a virus inoculum of concentration *V*_PFU_(0). For the SC, the starting time is *t*_start_ = −1h and the initial virus concentrations were fixed to *V*_PFU,SC_(−1h) = MOI ⋅ *c*_PFU_/(*β* ⋅ [1 − exp(−*c*_PFU_ ⋅ [1h])]) as per (Eq 4) for an MOI = 4, and *V*_RNA,SC_(−1h) = *p*2*r* × *V*_PFU,SC_(−1h), where *p*2*r* is a free parameter representing the ratio between total and infectious virus (*V*_RNA_/*V*_PFU_) within the initial experimental inoculum. In order to reproduce the sudden drop in viral titer brought about by the post-incubation experimental rinsing procedure in the SC experiment, we apply a simulated wash in our model at time *t* = 0 by reducing *V*_PFU_ and *V*_RNA_ by a fixed common factor (*W* = 8.0 × 10^−5^) across all strains. This wash parameter is determined by selecting the value that minimizes the total SSR across all strains and thus best represents the experimental drop in virus concentration caused by the rinsing procedure. For the MC, the starting time is *t*_start_ = 0, the initial infectious viral concentration is a free parameter, *V*_PFU,MC_(0), and the total virus concentration is fixed to *V*_RNA,MC_(0) = *p*2*r* × *V*_PFU,MC_(0). Leaving *V*_PFU,MC_(0) as a free parameter was necessary since setting it from the experimental MOI as was done for the SC experiment caused viral growth in the MC to occur in the model much sooner than was experimentally observed. This growth delay was also observed in our previous analysis [18].

As in previous work we define two separate infectious virus production rates for the SC and MC assays (*p*_PFU,SC_, *p*_PFU,MC_) experiments. This is to account for the observed ∼ 100-fold decrease in the infectious viral titer (*V*_PFU_) peak in the SC assay compared to that observed in the MC assay, whereas both SC and MC assays have similar total viral load (*V*_RNA_) peaks. This decrease—observed only in the infectious virus titer yield but not in the total virus yield—is thought to be due to the enhanced effect of defective interfering particles in the SC assay which suppress infectious virus production (*V*_PFU_) in favour of defective virus particles, leaving the total virus production (*V*_RNA_) unaffected [44, 45]. The model in Eq (1) also allows for variation of the number of compartments used to model the duration of the eclipse (*n*_*E*_) and infectious (*n*_*I*_) phases. The number of compartments in each phase impacts the standard deviation or spread (*σ*_*E*_, *σ*_*I*_) of the probability distribution for the time spent by a cell in that particular phase. We determined (not shown) that for the current set of data, the model in Eq (1) is insensitive to the specific values of *n*_*E*_ and *n*_*I*_, provided that these values are approximately > 50. Hence, we fixed *n*_*E*_ = *n*_*I*_ = 60.

Because we have made slight modifications to our model and our parameter definitions since our previous, published analysis of the H275Y NA mutation in the H1N1pdm09 strain background, this data is re-analyzed here under the current, revised model for comparative purposes. Consequently, some parameter names or expressions described here for this pair of strains (WT-H275 and MUT-H275Y) differ from that reported in [18]. Additionally, the total influenza virus concentration in the supernatant (*V*_RNA_) for the SC assay, which was unavailable at the time of our previously published analysis, has since been determined from frozen samples collected during the original experiment and combined here with the previous SC, MC and MY measurements to form a complete data set, like that collected for our current pair of strains (WT-I223 and MUT-I223V). Data for the WT-H275 and MUT-H275Y assay set shows a decay in measured total influenza virus concentration, *V*_RNA_, over time, as seen in Fig 5. Consequently, for these two strains a particle loss parameter (*c*_RNA_) was added to the model, and fixed via least-squares fitting of the data. For the WT-I223 and MUT-I223V assay set no such decay was observed and *c*_RNA_ was fixed to zero.

Given that the experimental measurements include a measure of experimental uncertainty and variability, we need to determine the probability distribution for each of our model’s parameters to reflect the experimental data, rather than a single set of misleading “best-fit” values for our parameters [46, 47]. In previous work, we used the bootstrap method to estimate these distributions, or rather the uncertainty of the parameter values given that of the experimental data [18]. Here instead of the bootstrap method, a Markov chain Monte Carlo (MCMC) approach is used to extract the posterior probability density distribution of our model’s parameters to match the spread in the experimental data, following the work of others [46–49]. Both the bootstrap and MCMC methods are commonly used identifying parameter values from experimental data, but we preferred the MCMC method as it does not rely on minimization algorithms (e.g., least-square fitting using Nelder-Mead Simplex or Levenberg-Marquardt methods). The odeint function of python scipy’s integrate module is used to solve Eq (1), and the python module emcee is used to perform the MCMC [39, 40].

Given all the parameter adjustments discussed above, a total of 9 free parameters remain—two parameters describing the initial conditions (*V*_PFU,MC_(0) and *p*2*r*) and 7 kinetic parameters (*τ*_*E*_, *τ*_*I*_, *c*_PFU_, *β*, *p*_PFU,MC_, *p*_PFU,SC_, *p*_RNA_)—whose probability density distribution is determined using the MCMC method. In our MCMC simulations, 300 walkers initially distributed uniformly or log-uniformly throughout the 9-dimensional parameter space perform a random walk through that space, accepting or rejecting randomly chosen steps with probability $\exp(-\text{SSR}\left(\vec{p}\right))$. Here SSR($\vec{p}$) is the SSR at the location of the proposed step $\vec{p}$, such that the lower the SSR at the proposed step’s destination, the higher the probability of accepting that step. The random walk is continued until parameter convergence is achieved (∼ 3,000 accepted steps for each walker for a total of ∼ 900,000 accepted parameter sets), as assessed using the interval-based coverage ratio diagnostics for each parameter [50]. Herein, we report the median and 95% confidence interval of these sets for each parameter. To determine whether the value of a particular parameter for strain *A* statistically significantly differs from that of strain *B*, we compute the *p*-value as determined by a two-sided Z-test using the test statistics

$$\begin{array}{c} \text{TS}=\frac{\mu_{A-B}}{\sigma_{A-B}} \end{array}$$ (5)

where *μ*_*A* − *B*_ and *σ*_*A* − *B*_ are the mean and standard deviation, respectively, of the difference between random pairs of accepted MCMC parameters for strains *A* and *B*. Since several parameters (for example *β* and *p*_PFU,MC_) follow a lognormal distribution, the difference of the log_10_ rather than the difference of the parameters themselves was used.

### Re-analysis of the WT-H275 and MUT-H275Y strains

Since the mathematical model and analysis approach we adopt here differs from that in our previous investigation of the WT-H275 and MUT-H275Y strains [18], with the inclusion of previously-unavailable RNA data, and because we wish to compare the effect of the H275Y mutation to that of the I223V mutation, we re-analyzed this pair of strains here using our new, modified model and approach. The results are presented in Table 5. As reported previously, we still find that the primary effects of the H275Y mutation is a significant reduction of the eclipse period, a decrease in the production rate, and a compensating increase virus infectiousness (previously reported as *R*_0_/*b*, but reported here as *β*). The distribution medians and standard deviations have changed from [18] likely due to differences in how we simulate virus inoculation in the MC assay. This demonstrates that our previous conclusions and parameter trends were not impacted by the small changes we made to our model and methodology.

**Table 5:** Viral kinetics parameters for H1N1pdm09 WT and MUT-H275Y*.

Parameter	WT-H275 Value [95% CI]	MUT-H275Y Value [95% CI]	(H275 vs. H275Y) Significance
Eclipse period, *τ*_*E*_ (h)	6.9 [6.0–7.7]	9.4 [8.4–10.3]	***p* < 0.001**
Infecting time, *t*_infect_ (min)	8.4 [5.3–12.9]	7.0 [4.5–10.6]	*p* = 0.58
Infectious lifespan, *τ*_*I*_ (h)	46 [37–54]	38 [28–46]	*p* = 0.17
Virion decay rate, *c*_PFU_ (h^−1^)	0.145 [0.116–0.176]	0.145 [0.117–0.175]	*p* = 0.99
Total prod. rate, *p*_RNA_ (RNA/cell/h)	7460 [4240–13100]	1770 [970–3390]	***p* < 0.001**
Infectious prod. rate, *p*_PFU_ (PFU/cell/h)	350 [170–750]	82 [38–190]	***p* = 0.01**
Virus infectiousness, *β* (mL/PFU/h)	3.0 × 10^−7^ [1.2–7.7]	1.8 × 10^−6^ [0.8–4.2]	***p* = 0.005**
Inoculum infectiousness, $\frac{V_{\text{PFU}}(0)}{V_{\text{RNA}}(0)}$, $\left(\frac{\text{PFU}}{\text{RNA}}\right)$	2.7 × 10^−7^ [0.7–10.7]	5.0 × 10^−7^ [1.6–15.6]	*p* = 0.50
Multiplicity of infection (MOI)	4.2 × 10^−8^ [1.3–13.7]	7.9 × 10^−7^ [2.6–24]	***p* < 0.001**
Prod. infectivity ratio, $\frac{p_{\text{PFU,SC}}}{p_{\text{PFU,MC}}}$	2.4 × 10^−4^ [0.9–6.0]	2.9 × 10^−4^ [1.1–8.2]	*p* = 0.75

* Median parameter values for H1N1pdm09 WT-H275, MUT-H275Y.

### Parameter selection in simulating competition experiments

The parameters used for the simulated competition experiments between the WT-I223 and MUT-I223V strains are the median values reported in Table 1. To determine whether one strain has a statistically significant competitive advantage over the other, either in terms of infectious or total virus peaks or fraction of cells infected, we use the accepted walker positions from our MCMC analysis. Each individual walker position represents a set of accepted values for the 9 model parameters, that together reproduce strain behaviour as observed in the experimental assays. For each strain, we randomly select a walker position from its set of MCMC chains (out of a total of 300 × 3000 possible positions). We then perform a simulated competition experiment using these two parameter sets (one for WT-I223 and one for MUT-I223V), solving the ODE model to extract values for the peak infectious viral titer, total viral titer, and fraction of cells in the infectious state. We then repeat this procedure a thousand times, using at each iteration a new pair of parameter sets randomly selected with replacement from all accepted walker positions for each strain. From these thousand competition simulations we extract the statistical range of peak total and infectious viral titer and fraction of cells infected for each strain. To quantify whether the recovered distributions of peak viral loads and fraction of cells infected are distinct between the two strains, we compute the significance of the results as *p*-values by using a two-sided Z test for their difference, as per Eq (5).

Finally, a direct competition simulation between the two single-mutants (MUT-H275Y and MUT-I223V) would be instructive to determine which has a greater relative fitness. Since the MCMC analysis for the MUT-H275Y and MUT-I223V was conducted on data from two distinct experimental assays, we first need to express both mutants as a fold-change from a single wild-type (the median WT-H275 values, detailed further in Table 5). As before, for each iteration a parameter set for MUT-I223V and MUT-H275Y is randomly selected from their respective MCMC walker chains. The MUT-I223V parameter values are then re-calibrated as a deviation from the WT-H275 median values. For example, for WT-H275 the median value of the infection lifetime is *τ*_*I*, H275_(50) = 46h, whereas for WT-I223 the median value is *τ*_*I*, I223_(50) = 28h (see Table 1). A randomly-chosen walker position for MUT-I223V may have *τ*_*I*_ = 21h which, in the competition experiment, would be converted as:

$$\begin{array}{c} \tau_{I}\left(\text{mod}\right)=\frac{\tau_{I}}{\tau_{I,I223}\left(50\right)}\times \tau_{I,H275}\left(50\right)=34.5h\;. \end{array}$$

In other words, in this particular case the MUT-I223V has a infection lifespan of only 0.75 of that of the median wild-type lifespan. Once the entire MUT-I223V parameter sets is recalibrated to the base WT-H275 median values we can then conduct the simulated competition experiment and directly compare the two mutants. Having done this, we performed our competition simulations and obtained the results presented in Table 6.

**Table 6:** Simulated competition experiments*.

WT-H275 vs. MUT-H275Y, Dose = 0				With Dose = 15 nM		
Comp. Strains	PFU/mL	RNA/mL	Inf. frac.	PFU/mL	RNA/mL	Inf. frac.
WT-H275	1.3 × 10^9^	5.0 × 10^10^	0.56	1.1 × 10^4^	4.1 × 10^5^	0.00
MUT-H275Y	2.4 × 10^8^	8.7 × 10^9^	0.44	5.3 × 10^8^	1.9 × 10^10^	1.00
Significance	*p* = 0.06	***p* = 0.04**	*p* = 0.43	***p* < 0.001**	***p* < 0.001**	***p* < 0.001**
WT-I223 vs. MUT-I223V, Dose = 0				With Dose = 15 nM		
Comp. Strains	PFU/mL	RNA/mL	Inf. frac.	PFU/mL	RNA/mL	Inf. frac.
WT-I223	5.4 × 10^7^	1.5 × 10^10^	0.96	4.5 × 10^4^	1.3 × 10^7^	0.03
MUT-I223V	3.9 × 10^6^	5.2 × 10^8^	0.04	1.3 × 10^7^	1.9 × 10^9^	0.84
Significance	***p* < 0.001**	***p* = 0.002**	***p* < 0.001**	***p* < 0.001**	***p* < 0.001**	***p* < 0.001**
MUT-H275Y vs. MUT-I223V, Dose = 0				With Dose = 15 nM		
Comp. Strains	PFU/mL	RNA/mL	Inf. frac.	PFU/mL	RNA/mL	Inf. frac.
MUT-H275Y	5.4 × 10^8^	1.9 × 10^10^	0.98	5.3 × 10^8^	1.9 × 10^10^	0.999
MUT-I223V	1.2 × 10^8^	5.9 × 10^9^	0.02	4.4 × 10^5^	2.2 × 10^7^	0.001
Significance	***p* = 0.04**	*p* = 0.08	***p* < 0.001**	***p* < 0.001**	***p* < 0.001**	***p* < 0.001**

* Three sets of paired experiments were simulated. Significance of simulated peak viral titers or fraction of cells infected are provided in *p*-values.
